# The Lost Neural Hierarchy of the Autistic Self—Locked-Out of the Mental Self and Its Default-Mode Network

**DOI:** 10.3390/brainsci11050574

**Published:** 2021-04-29

**Authors:** Fuxin Lian, Georg Northoff

**Affiliations:** 1 Institute of Psychological Sciences, School of Education, Hangzhou Normal University, Hangzhou 311121, China; fxlian@hznu.edu.cn; 2 Institute of Mental Health Research, University of Ottawa, Ottawa, ON K1Z 7K4, Canada

**Keywords:** mental-self, self-reference, autism spectrum disorder, default-mode network, predictive coding, weaken central coherence, theory of mind

## Abstract

Autism spectrum disorder (ASD) is characterized by a fundamental change in self-awareness including seemingly paradoxical features like increased ego-centeredness and weakened self-referentiality. What is the neural basis of this so-called “self-paradox”? Conducting a meta-analytic review of fMRI rest and task studies, we show that ASD exhibits consistent hypofunction in anterior and posterior midline regions of the default-mode network (DMN) in both rest and task with decreased self–non-self differentiation. Relying on a multilayered nested hierarchical model of self, as recently established (Qin et al. 2020), we propose that ASD subjects cannot access the most upper layer of their self, the DMN-based mental self—they are locked-out of their own DMN and its mental self. This, in turn, results in strong weakening of their self-referentiality with decreases in both self-awareness and self–other distinction. Moreover, this blocks the extension of non-DMN cortical and subcortical regions at the lower layers of the physical self to the DMN-based upper layer of the mental self, including self–other distinction. The ASD subjects remain stuck and restricted to their intero- and exteroceptive selves as manifested in a relative increase in ego-centeredness (as compared to self-referentiality). This amounts to what we describe as “Hierarchical Model of Autistic Self” (HAS), which, characterizing the autistic self in hierarchical and spatiotemporal terms, aligns well with and extends current theories of ASD including predictive coding and weak central coherence.

## 1. Introduction

Autism spectrum disorder (ASD) is a complex psychiatric condition that is characterized by multiple symptoms. Cognitive symptoms like changes in autobiographical/episodic memory [1,2,3,4] are coupled with deficits in social cognition as in theory of mind [5,6,7], affective changes including emotion, empathy, and facial expression [8,9,10], hypersystemizing [11], motor symptoms like difficulty of action imitation [12,13,14], stereotypies and repetitions [15,16,17], and multimodal sensory integration [18,19,20,21,22]. Yet, on a deeper level beneath the various functions, an altered sense of self, i.e., self-awareness, has been described and is a key disturbance of autism [23].

In their original descriptions of autism, Kanner (1943) and Asperger (1944) point out a fundamental or basic disturbance of self: the self in ASD is only himself and self-sufficient [24] and feels neither an integral part of the world nor stands in a lively dynamic relationship with its environment [25]. More recently, Lombardo and Baron-Cohen (2010, 2011) highlighted what they describe as “paradox of self” in ASD [23,26] (see also [27,28,29]). On the one hand, the autistic self is highly centered on itself, showing an abnormally high degree of ego-centeredness as manifest in social isolation and loneliness, inability to read the emotions, feelings, and facial expressions of others [30,31,32], and major deficits in social cognition like theory of mind [5,6,7]. Such high ego-centeredness is, on the other hand, contrasted by weak self-referentiality with decreased use of “I” in language [33,34,35], no mention of own internal states, e.g., own emotion [10,36,37], own theory of mind [27,38,39], changes in time processing like duration estimation of shorter and longer time intervals as deficits in connecting different time points [40,41,42], decreased introspection [43,44], decrease in interoception [45,46,47], and reduced autobiographical memory [1,2,3,4] (see though Markram and Markram, 2012 [48], as well as Lind et al., 2020 [49]).

How is it possible that seemingly two contradictory features like increased ego-centeredness and decreased self-referentiality can co-occur within one and the same person’s self? Lombardo and Baron-Cohen (2010) assume a shared deficit in the neural circuitry that encodes self-representation, including self–other distinction and self–other awareness [26]. Various imaging studies, most often using fMRI, investigated self-reference in ASD. They observed changes in various anterior and posterior regions of the default-mode network (DMN) and outside the DMN during self-referential tasks (see below). At the same time, resting state abnormalities could also be observed in anterior and posterior DMN (and also non-DMN) of ASD, which again showed major changes in DMN (see for recent review Lau et al., 2020 [50] and below). Given that various findings in healthy subjects indicate neural overlap of self and DMN resting state [51,52,53,54,55,56], one may assume a close relationship of resting state and self-specific task-related changes in DMN of ASD. The goal of the present paper is to review recent fMRI findings in ASD during both rest and self-referential task states in order to reconcile the seemingly features of increased ego-centeredness and decreased self-referentiality.

A recent meta-analytic study on the self in healthy subjects suggests a multilayered nested hierarchical model of self with interoceptive self, exteroceptive self, and mental self: neural correlates range from subcortical regions and insula (interoceptive self) over medial prefrontal cortex and temporo-parietal junction (TPJ) (exteroceptive self) to anterior and posterior DMN (mental self) [57,58]. Following such nested neural hierarchy of self, we propose that DMN hypofunction in both rest and task states (decreased/absent self–non-self differentiation) renders it impossible for ASD subjects to access the most upper layer of their self, the mental self. This results in the weakening of their mental self with decreases in both self-referentiality and self–other awareness—they are locked-out of their mental self, including its self–other distinction. While, at the same time, they remain restricted to the lower layers of the intero- and exteroceptive self as mediated by non-DMN and subcortical regions—this is manifested in increased ego-centeredness. We conclude that DMN hypofunction in ASD disrupts the neural hierarchy of self as it blocks the mental self, i.e., it is locked-out, which weakens self-referentiality and, at the same time, (relatively) increases the lower layers of the intero- and exteroceptive self, i.e., increased ego-centeredness. Following these meta-analytic observations, we postulate a novel model of self in ASD, a “Hierarchical Model of Autistic Self” (HAS).

## 2. Materials and Methods

Studies included in this review were collected from the database PubMed with a time frame up to October 2020. The procedure included two different stages: collecting neural studies with self-relative tasks and resting state fMRI studies. Due to the heterogeneity of studies in terms of subject, measures, and method as well as the breath of data we included, we refrained from quantitative meta-analysis and focused on narrative review.

### 2.1. Collecting Neural Studies with Self-Relative Tasks

In this stage, fMRI studies using physical or psychological self-relative tasks were searched. The physical self-relative tasks included heartbeat, own-face/body recognition, agency, ownership of body or objects, and self-initiated motion. For these tasks, search terms used were “heartbeat”, “own face”, “self-face”, “own body”, “self-body”, “ownership”, “agency”, and “executed action”. The psychological tasks covered own-name recognition, self-emotion processing, autobiographical memory, trait adjective judgement, self-relevance sentence judgment, and self judgement in other complex tasks (i.e., decision making or pseudo-interactive joke). Search terms for these tasks were “self-name”, “own-name”, “self-reference”, “self”, “self-emotion”, “autobiographical memory”, and “episodic memory”. All the search terms were combined with “autism” and “fMRI” to included temporal and functional neural studies on ASD.

A total of 324 results for fMRI were obtained with the initial search. Then, the results were screened by reviewing the title and abstract carefully and, if necessary, the whole paper was read through. Only studies that met the following criteria were include for further analysis: (a) fMRI studies; (b) using at least one of the physical self-relative tasks or psychological self-relative tasks; (c) including both individuals with ASD and TD group as participants. Those studies with narrative-qualitative reviews and meta-analysis were excluded.

Finally, only 19 fMRI studies on this topic were included. With intensive reading, we extracted main information including demographic characteristic of participants (i.e., age bracket), self-relative tasks, and abnormal activity of individuals with ASD contrasted to TD group for fMRI studies (Table 1).

### 2.2. Collecting Resting-States fMRI Studies

To verify the hypothesis that abnormal neural activity of individuals with ASD during self-relative tasks could be mapped into the neural activation during resting states, resting state fMRI studies were searched in this stage with the following terms: “autism”, “resting sates”, and “fMRI” in PubMed.

After initial research, 472 results were obtained. A screening procedure as a last stage was conducted to assess whether the studies (a) were resting state fMRI studies; (b) included both individuals with ASD and TD group as participants; (3) reported neural activation or functional connectivity of any brain regions mentioned in results of self-relative task reviewing parts. Those studies with narrative-qualitative reviews and meta-analysis were also excluded.

Finally, 97 studies were gained, from which the demographic characteristic of participants (i.e., age bracket), fMRI data processing methods, and abnormal neural activation or functional connectivity of individuals with ASD contrasted to TD group were extrapolated (Table 2).

With these two reviewing parts, we were able to compare the neural activity during self-relative tasks to resting state fMRI and then make hypotheses about the changes in activity from task to rest and how they can be intrinsically related.

## 3. Results

### 3.1. Task fMRI in ASD: Task-Related Neural Activity and Functional Connectivity during Self

**Figure 1:**
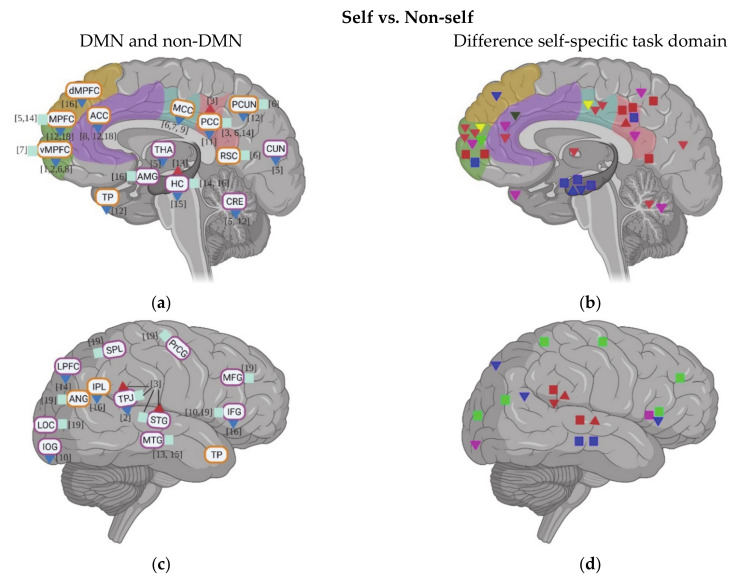

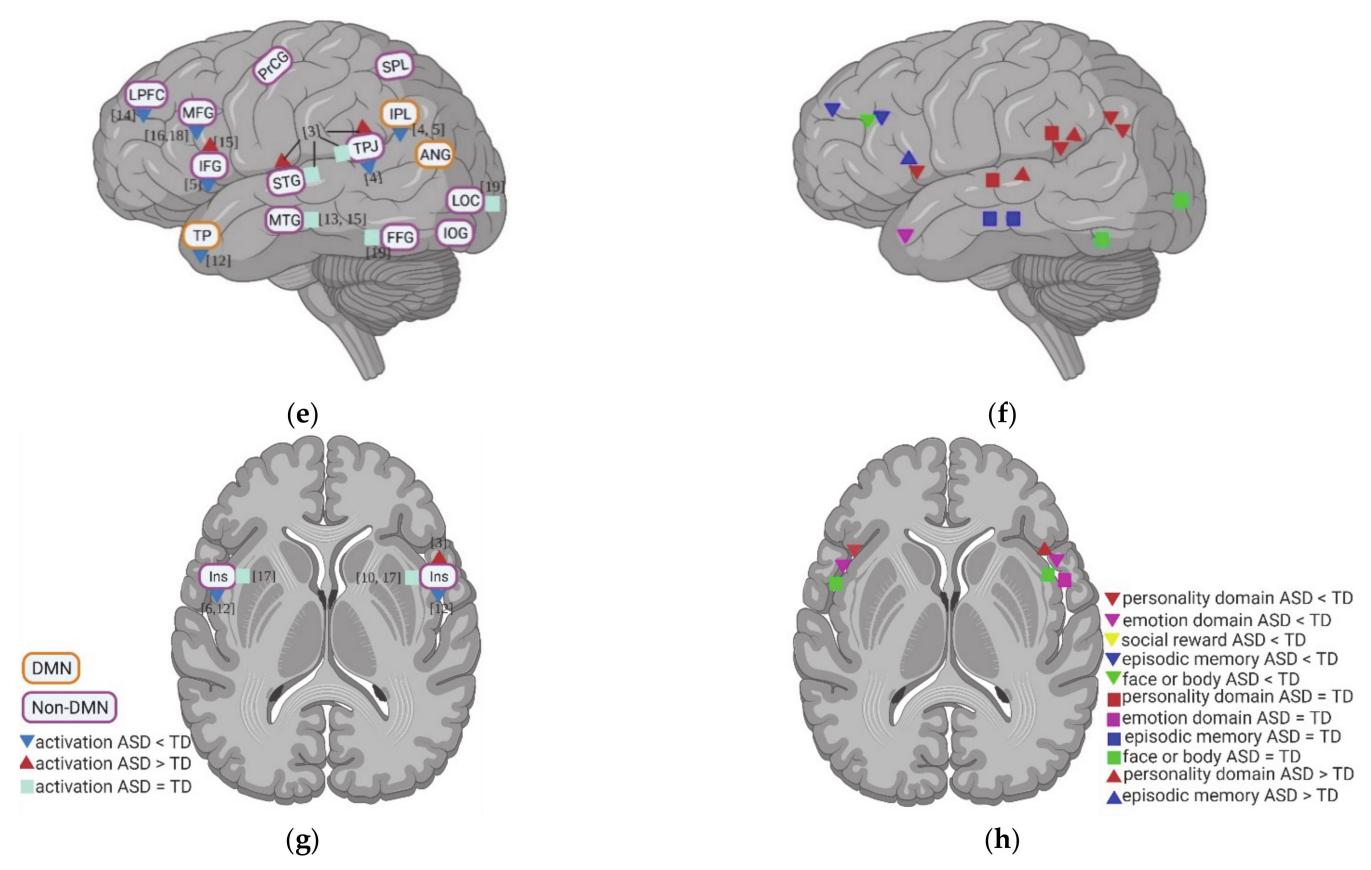
Activation of DMN and non-DMN regions for individuals with ASD compared to TD during self-specific tasks in different studies (left side) and different domains (right side): (**a**,**b**) DMN, subcortical regions and cerebellum (CRE); (**c**,**d**) related regions in right lateral hemisphere; (**e**,**f**) related regions in left lateral hemisphere; (**g**,**h**) insula (Ins). The numbers in the figure correspond to the study numbers in Table 1.

A total of 19 studies evaluated the neural activity with self-specific tasks in individuals with ASD. Among these studies, 16 studies focused on the psychological self (7 studies with the self-reference task, 2 studies with social reward task, 3 studies with emotional task, and 4 studies with episodic memory task), while the other studies focused more on the physical self (1 study about heartbeat and 2 studies with self-body recognition task) (Table 1). Across these different tasks, decreased activation in DMN regions during self-specific tasks/stimuli was reported, with a particular emphasis on reduction in medial prefrontal cortex (MPFC) (including ventral medial prefrontal cortex (vMPFC) and dorsal medial prefrontal cortex (dMPFC)), anterior cingulate cortex (ACC), media cingulate cortex (MCC), and inferior parietal lobule (IPL) (Figure 1a–f). In contrast to the anterior midline regions, findings in posterior cingulate cortex (PCC)and precuneus (PCUN) are not as consistent among different studies and tasks: decreased activity was observed during the emotional recognition tasks in ASD, whereas they did not show any abnormalities in these regions during the self-reference task (Figure 1b).

In non-DMN regions, hypoactivity of cerebellum (CRE) and middle frontal gyrus (MFG) is reported for individuals with ASD compared to TD in a few studies (Figure 1c–f). Contradictory results were found for the activity of inferior frontal gyrus (IFG) (Figure 1c–f), TPJ (Figure 1c–f), insula, and hippocampus (HC) across different tasks (Figure 1g,h).

**Figure 2:**
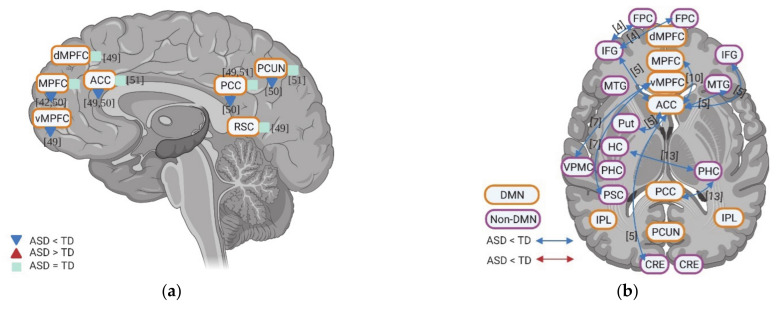
(**a**) Deactivation of DMN for individuals with ASD compared to TD during self-specific tasks in contrast with resting states. The numbers in the figure correspond to the study numbers in Table 2. (**b**) Functional connectivity within DMN and between DMN and non-DMN regions for individuals with ASD compared to TD during self-specific tasks. The numbers in figure correspond to the study numbers in Table 1.

Few studies also evaluated the task-related deactivation of DMN in ASD. In anterior regions of DMN, individuals with ASD show either reduced deactivation (e.g., vMPFC, MPFC, and ACC) or “normal” levels of deactivation (e.g., ACC and dMPFC). Although one study reports reduced deactivation in PCC and PCUN in ASD, two studies could now show group difference in deactivation of PCC, PCUN, and retrosplenial cortex (RSC) (Figure 2a).

Finally, few studies investigated the functional connectivity among different brain regions during self-specific tasks. With the few results available, hypoconnectivity between DMN regions (e.g., vMPFC, ACC, and PCC) and non-DMN regions (e.g., IFG, medial temporal gyrus (MTG), CRE, putamen (Put), parahippocampal cortex (PHC), ventral premotor cortex (VPMC), and primary somatosensory cortex (PSC)), as well as within the DMN itself (e.g., MPFC-ACC) were reported (Figure 2b).

Taking together, unlike the inconsistent results of most of the non-DMN regions, individuals of ASD show decreased activity, i.e., amplitude or magnitude in DMN, especially in the anterior parts across different self-specific tasks, i.e., domain-general. Decreased self-specific activity in these regions is further supported by observations that individuals of ASD also exhibit reduced deactivation and hypoconnectivity within DNM and of DMN with non-DMN regions during various self-specific tasks.

### 3.2. Resting State fMRI in ASD: Resting State Functional Connectivity (rsFC) within DMN

**Figure 3:**
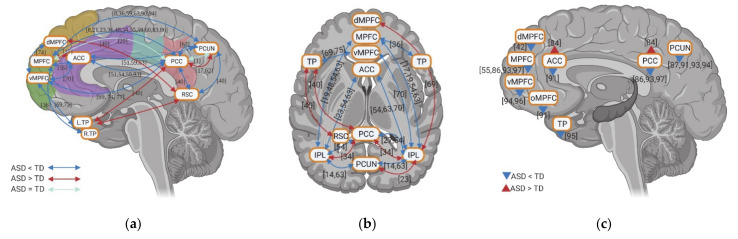
Inter-regional functional connectivity (**a**,**b**) and whole-brain resting state functional connectivity (**c**) of DMN for individuals with ASD compared to TD. The numbers in the figure correspond to the study numbers in Table 2.

The fMRI studies investigating rsFC in individuals with ASD highlight abnormal activity within DMN. Although few studies showed hyperconnectivity of dMPFC with RSC and right IPL, left temporal pole (TP) with RSC and PCC, a decrease in rsFC between anterior DMN (e.g., MPFC/vMPFC and ACC) and posterior DMN (e.g., PCC, PCUN, and IPL) is a consistent finding across various studies (Figure 3a,b). Within anterior DMN regions, hypoconnectivity among vMPFC, dMPFC, ACC, and bilateral TP was observed, with the exception of one of study that reported hyperconnectivity between the MPFC and ACC (Figure 3a,b). Within the posterior DMN regions, both hypoconnectivity and hyperconnectivity between PCC and PCUN, PCC and bilateral IPL, and PCUN and bilateral IPL has been observed by a few studies, while decreased rsFC was reported between RSC and PCC, PCUN in one study. When it comes to the whole-brain rsFC, both anterior and posterior DMN regions show decreased rsFC (with only one study showing increased rsFC in ACC and PCC) (Figure 3c).

**Figure 4:**
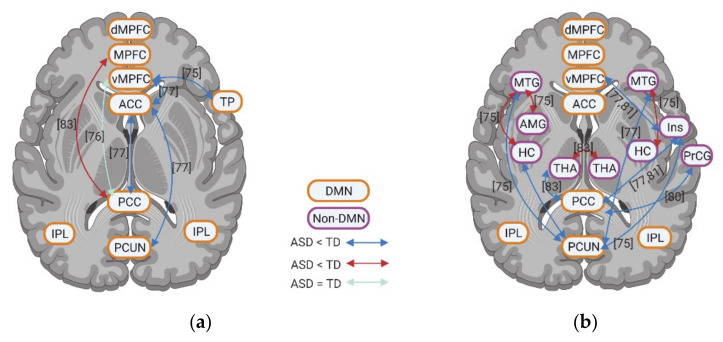
Dynamic functional connectivity between regions within DMN regions (**a**) as well as between DMN and non-DMN regions (**b**) for individuals with ASD compared to TD. The numbers in the figure correspond to the study numbers in Table 2.

Unlike the results of static rsFC, the few studies on dynamic rsFC show contradictory rsFC results between anterior DMN (vMPFC and ACC) and posterior DMN (PCC and PCUN) in ASD including weaker, equal, and stronger rsFC, respectively (Figure 4a). A few studies investigating the dynamic whole-brain rsFC of DMN also report contradictory results, with two studies showing increased rsFC and two studies reporting decreased rsFC for individuals with ASD. More consistent is a decrease of dynamic rsFC within anterior DMN (e.g., vMPFC, ACC, and TP) in ASD (Figure 4a).

Overall, individuals with ASD show decreased static rsFC between anterior DMN and posterior DMN as well as reduced whole-brain hypoconnectivity of the DMN. In contrast, results on dynamic rsFC are more inconsistent, though they consistently also show decreased variability in anterior DMN rsFC.

### 3.3. Resting State fMRI in ASD: Resting State Functional Connectivity between DMN and Non-DMN

**Figure 5:**
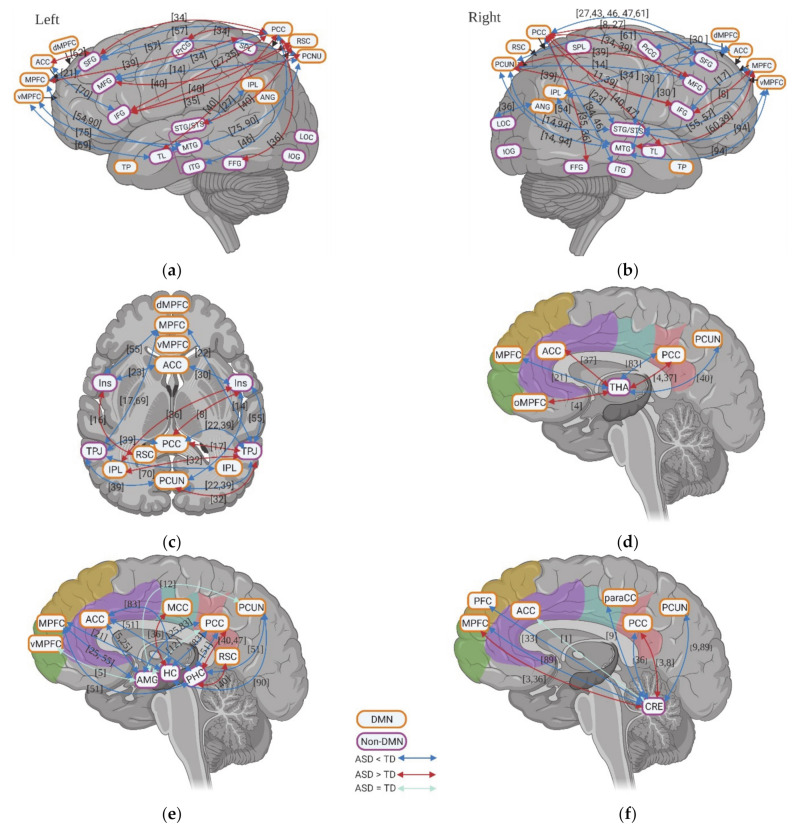
Functional connectivity between DMN and non-DMN regions for individuals with ASD compared to TD. In this figure, (**a**,**b**) shows the functional connectivity between DMN and non-DMN regions in left and right hemisphere; (**c**) shows functional connectivity between DMN and Insular (Ins), temporo-parietal junctions (TPJ); (**d**) shows the functional connectivity between thalamus (THA) and DMN; (**e**) shows the functional connectivity between DMN and amygdala (AMG), hippocampus cortex (HC), and parahippocampal cortex (PHC); (**f**) shows the functional connectivity between cerebellum (CRE) and DMN. The numbers in the figure correspond to the study numbers in Table 2.

The various resting state fMRI studies examined also underline altered rsFC between DMN and non-DMN regions in individuals with ASD. In the lateral frontal regions (Figure 5a,b), superior frontal gyrus (SFG) especially in the right hemisphere is reported to have altered rsFC with posterior DMN (e.g., PCC, PCUN, and IPL) with a tendency towards hypoconnectivity: six studies report decreased rsFC, while three studies observed increased rsFC. IFG showed hyperconnectivity with posterior DMN (e.g., PCC, PCUN, and RSC), and MFG also exhibited predominant increased rsFC with posterior DMN: three studies show increased rsFC and two studies report decreased rsFC. Unlike posterior DMN, there are less studies investigating the rsFC between anterior DMN and lateral frontal regions. For IFG, weaker rsFC with anterior DNM was found in two studies while stronger rsFC was found in one study. For SFG, both decreased and increased rsFC with anterior DMN were found in one study. In temporal lobe (TL) (Figure 5a,b), studies reported hyperconnectivity with PCC when considering TL as whole region. However, different subregions of temporal lobe showed distinct rsFC patterns with posterior DMN. Hypoconnectivity was observed for superior temporal gyrus (STG) with PCUN and IPL, MTG with PCUN and IPL, and inferior temporal gyrus (ITG) with PCC, whereas hyperconnectivity was reported for fusiform gyrus (FFG) with PCC. As opposed to the posterior DMN, the anterior DMN, especially the MPFC/vMPFC, exhibits relatively consistent hypoconnectivity with temporal lobe subregions, including decreased rsFC for the whole TL, a decrease in rsFC for STG, and weaker rsFC for MTG, but also sees increased rsFC MTG. There is also hypoconnectivity of TPJ with both anterior, i.e., MPFC, and posterior DMN, i.e., PCC, PCUN, and IPL (Figure 5c). Finally, bilateral insula was observed to have decreased rsFC with anterior DMN (MPFC and ACC) and increased rsFC with posterior DMN (PCC, RSC and IPL); there is also higher rsFC with PCUN (Figure 5c).

Compared to the static rsfMRI studies, dynamic rsfMRI studies showed relatively consistent weaker rsFC of DMN, especially the posterior DMN with cortical non-DMN regions, i.e., MTG, precentral gyrus (PrCG), and insula (Figure 4b).

In general, individuals with ASD showed decreased rsFC between SFG and posterior DMN regions as well as hypoconnectivity of STG, MTG, ITG, TPJ, and insular with both anterior and posterior DMN during resting states. Weaker dynamic rsFC of DMN with non-DMN regions was also found for individuals with ASD. However, they had predominant hyperconnectivity of IFG, MFG, and FFG with posterior DMN during resting states.

### 3.4. Resting State fMRI in ASD: Subcortical–Cortical Resting State Functional Connectivity

Subcortical regions also had changes in their rsFC with DMN. The amygdala (AMG) exhibits hypoconnectivity with both anterior (e.g., MPFC and ACC) and posterior DMN (e.g., PCC) for individuals with ASD; although some studies did not show any group difference (Figure 5e). Similar to AMG, HC shows weaker rsFC both with anterior (e.g., MPFC and ACC) and with posterior DMN (e.g., PCC and PCUN) with the exception of higher rsFC with MCC (Figure 5e). However, PHC exhibits decreased rsFC with anterior DMN whereas it showed increased rsFC with posterior DMN (e.g., PCC and RSC) (Figure 5e). Few studies focused on thalamus (THA) rsFC with DMN and showed contradictory results: two studies observed increased rsFC with ACC/orbital MPFC and PCC, while one reports decreased rsFC with MPFC, PCC, and PCUN (Figure 5d). Furthermore, the cerebellum was observed to have increased rsFC with MPFC and PCC, whereas it shows decreased rsFC with PCUN (Figure 5f). Finally, in contrast to the static rsfMRI studies, dynamic rsfMRI studies showed relatively consistent weaker rsFC of DMN, especially the posterior DMN with subcortical regions, i.e., HC and THA (Figure 4b).

In sum, AMG and HC show hypoconnectivity with both anterior and posterior DMN in static and dynamic rsFC. In contrast, the CRE exhibits predominant hyperconnectivity with DMN during resting states. The THA shows a contradictory rsFC pattern across the studies.

### 3.5. Resting State fMRI in ASD: ALFF and REHO in DMN Regions

**Figure 6:**
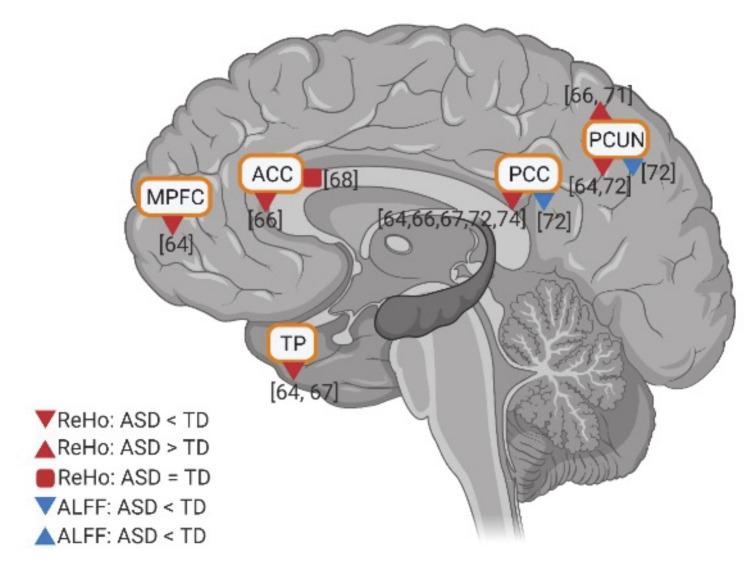
Intraregional resting state activity (ALFF and REHO) in DMN for individuals with ASD compared to TD. The numbers in the figure correspond to the study numbers in Table 2.

Few studies investigated the intraregional resting state activity using amplitude of low-frequency fluctuations (ALFF) and regional homogeneity (ReHo), in individuals with ASD compared to TD controls. In these studies, relatively consistent results of the intraregional activity of the DMN were reported. Anterior DMN regions (e.g., MPFC, ACC, and TP) have reduced activity in ReHo. Meanwhile, although two studies reported hyperactivity in PCUN in ReHo, the posterior DMN regions (e.g., PCC and PCUN) show decreased activity in both ALFF and ReHo in ASD (Figure 6). In general, findings show decreased intraregional resting state activity in anterior and posterior DMN regions in ASD.

## 4. Discussion

How can increased ego-centeredness in ASD co-occur with weakened self-referentiality? Recent fMRI studies reveal decreased if not absent neural differentiation of self- and non-self-reference in anterior and posterior midline structures of the DMN. The same DMN regions also show decreased resting state connectivity among each other while, at the same time, being abnormally connected to non-DMN regions like lateral prefrontal, temporal, hippocampus/amygdala, and cerebellum.

Presupposing a neural hierarchical model of self, we propose that access to the mental self as DMN-based upper layer of self is blocked in ASD—this weakens self-referentiality including self-awareness and self–other distinction. That, in turn, shifts the focus to the lower layers of self, the intero- and exteroceptive self as mediated by non-DMN cortical and subcortical regions, resulting in the (relative) increase of ego-centeredness. Accordingly, the neural hierarchy of self is disrupted in ASD as its most upper layer, the DMN-based mental self, is hypofunctional and blocked—they are locked-out of their own mental self. The assumption of such “Hierarchical model of Autistic Self” (HAS) does not only explain the self-paradox but also aligns well and extends current theories of ASD like predictive coding and central coherence.

### 4.1. Hypoactivity and -Connectivity within Midline DMN I—Weakening of Self-Referentiality or “Locked-out of Their Own Mental Self”

One of the most consistent observations in ASD is the lack of self-referentiality on both psychological and neural grounds. Various facets of the so-called psychological self [59] like use of the term “I” [33,34,35], mention of internal states [27,38,39], reference to own emotions and feelings [10,36,37], introspection [43,44], and episodic memory [1,2,3,4] are decreased in ASD [23,26,29,60]. Neuronally, the various task studies on self-specificity show decreased self–non-self differentiation in anterior and posterior midline structures of the DMN holding across different modalities and domains, i.e., supra-modal and domain-general. Given the close link of midline DMN and self-referentiality in healthy subjects [54,58,61,62,63,64,65], reduced midline DMN-based self–non-self differentiation is rather likely to be related to the decreased self-referentiality and hence the weakened mental self in ASD.

In addition to its reduced task-related activity during self-reference, midline DMN activity is also less connected between its anterior and posterior midline regions, as shown in both task and rest functional connectivity. Especially, reduced anterior–posterior midline DMN resting state functional connectivity is an often-observed finding [66,67,68,69,70,71,72] and confirmed in recent quantitative meta-analysis [50]. This suggests that even in the resting state, self-reference of especially internally oriented inputs and cognition is also impaired in ASD. Specifically, based on healthy subject data [54,56,58,65,73,74,75], we assume that the autistic self is already altered during the more internally oriented cognition dominating in the resting state; this is carried over to subsequent task states where more externally oriented cognition dominates, such as during tasks requiring self-referential cognition. Accordingly, DMN hypoconnectivity—and activity—renders ASD subjects unable to access their own mental self during both rest and task states—they are locked-out of their own mental self.

### 4.2. Hypoactivity and -Connectivity within Midline DMN II—Weakening of Internally Oriented Cognition

The midline DMN also mediate other forms of internally oriented cognition like episodic simulation or mental time travel [76,77,78], mind-wandering [79,80], social cognition including theory of mind [81,82,83,84], and autobiographical memory [82,85,86,87]. While autobiographical memory and social cognition/theory of mind have received coverage showing well-known deficits in ASD, the situation is less clear for mental time travel, that is, episodic simulation of past, present, and future. Data in healthy subjects show strong association of episodic simulation with midline DMN [76,77,78]. One would consequently expect DMN hypoconnectivity in ASD to lead to deficits in episodic simulation, which has indeed been observed in some psychological studies [2,88,89].

How can DMN hypoactivity and connectivity impair the ASD subjects’ ability of mental time travel of their own self? We tentatively hypothesize that the neuronal desynchronization between anterior and posterior midline DMN regions, as indexed by their reduced functional connectivity in both rest and task, may make it impossible for them to virtually expand their present self into both past and future on the mental level: the lower the anterior–posterior midline functional connectivity, the lower the degree of their synchronization, and the less subjects may mentally be able to virtually expand their present self into past and futures selves (as that requires expansion of neural activity across both anterior and posterior midline DMN) [76,77,78]. Such anterior–posterior DMN-based decreased temporal expansion into past and future selves is well compatible with both decreased midline DMN rsFC as well as phenomenological reports of increased interruption and desynchronization of past and future with the present self in ASD [90,91,92,93].

Other forms of internally oriented cognition like mind-wandering, social cognition, and autobiographical memory retrieval may also be affected by desynchronization of anterior–posterior midline DMN. For instance, mind-wandering and its spontaneous thoughts [94] are well known to recruit midline DMN regions as standing in a balance with lateral prefrontal cortex, i.e., central executive network (CEN) [79,80,95]. If the DMN is hypoactive during both rest and task, the DMN–CEN balance is also abnormal, as it has indeed been shown in ASD [67,96,97,98]. On a more cognitive level, DMN–CEN disbalance towards the dorsolateral prefrontal cortex may result in abnormally increased rationality and systematizing in cognition, i.e., hypersystematizing [11]. In contrast, motivation/reward and affective components of the self as mediated by especially anterior DMN [99,100,101] may be suppressed in both internally and externally oriented thoughts, as is indeed typical for ASD [11]. Finally, social cognition is also well known to be mediated by midline DMN in conjunction with TPJ and other temporal regions [83,102,103,104]. Decreased connectivity and thus reduced synchronization among these regions may again impair the integration of the various internal and external inputs and ultimately block access to the cognition of the other on a mental level, including its distinction from the own mental self.

Taken together, the reduced activity and connectivity of anterior and posterior midline DMN carries major consequences for not only the self, i.e., reduced self-referentiality with weakened mental self, but also for various forms of internally oriented cognition. In the same way that access to the mental self is diminished, access to other forms of internally oriented mental activity is also most likely reduced, including mental time travel/episodic simulation, the balancing of affective and cognitive components within the self and its thoughts, and social cognition with self–other distinction. Decreased anterior and posterior midline DMN may thus lock-out the autistic subjects from the internally oriented cognition of their own mental self—both self and internally oriented cognition no longer show self-expansion or self-prioritization [105,106,107].

### 4.3. Abnormal DMN–Non-DMN and Subcortical–Cortical Topography—Increased Ego-Centredness

In addition to the within-DMN task and rest changes, the DMN is also abnormally connected to various non-DMN regions in ASD. This includes a differential connectivity pattern of anterior and posterior midline DMN to temporal cortex, prefrontal cortex, and subcortical regions like AMG, HC, and PHC, and CRE. Although not fully consistent, the findings converge on decreased anterior DMN connectivity to prefrontal and temporal cortical regions while posterior midline DMN seemingly exhibits the opposite pattern, namely, increased connectivity. Moreover, functional connectivity of anterior and posterior midline DMN to subcortical AMG, HC, and PHC is mostly decreased, whereas the DMN is too strongly connected with CRE. Together, these findings, albeit tentative due to some inconsistencies, suggest an overall change in the brain’s topography with altered DMN–non-DMN balances on the cortical level and altered subcortical–cortical balance.

How is such potentially altered subcortical–cortical and DMN–non-DMN topography related to the paradox of the autistic self? A recent large meta-analysis in healthy subjects suggests a multilayered, nested hierarchical model of self [58]. There is a basic or lower layer of self, an interoceptive self that is related to regions that mostly process interoceptive stimuli from the own body, that is, insula, dorsal ACC, THA, and PHC, thus including mainly regions of the salience network [58,108]. The next or middle layer of self includes what Qin et al. (2020) describe as exteroceptive self that recruits insula, interior frontal gryus, premotor cortex, TPJ, and MPFC: these regions process exteroceptive and proprioceptive inputs, thus extending beyond the own body to the social world [58]. Finally, there is the most upper layer of the mental self that recruits all of the above-mentioned regions as well as more extensive anterior and posterior midline DMN regions—as this is mainly induced during typical self-referentiality paradigms, the authors speak of a mental self [58]. Since the regions of the lower layers all resurface in and are complemented by additional regions in the upper layers, Qin et al. (2020) speak of a nested neural hierarchical model of self with the interoceptive self as the bottom and the mental self at the top of the hierarchy [58].

**Figure 7:**
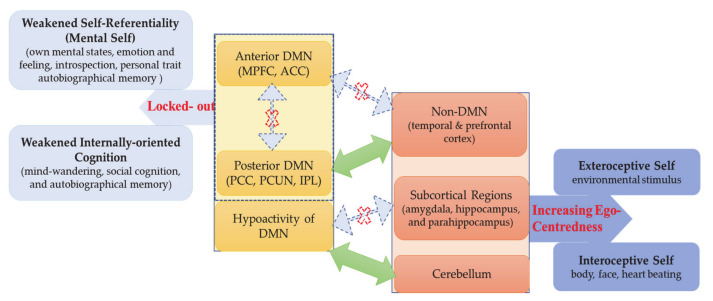
Hierarchical Model of Autistic Self (HAS): locked-out mental-self and increased ego-centeredness in individuals with ASD.

How does this neural multilayered nested hierarchical model of self stand in relation to the self in ASD? The findings are clear. The uppermost layer of the mental self and its DMN at the top of the hierarchy are impaired and blocked for the ASD subject, who is thus locked-out from both its DMN and mental self. Albeit not fully consistent, the resting state connectivity pattern suggests that non-DMN regions like the temporal lobe and the subcortical regions stand in abnormal balance to the anterior and posterior midline DMN. As these abnormally connecting non-DMN and subcortical regions include some of those implicated in intero- and exteroceptive layers of self, we assume that the brain’s topography is restricted towards processing intero- and exteroceptive processing layers of self (or bodily and environmental self, as described by Qin et al. 2020) while its DMN-based mental layers remain blocked [58] (see Figure 7).

This may shift the hierarchy and its balance: the intero- and exteroceptive self, i.e., physical self, may become isolated from their mental realization, including self-awareness and self–other distinction. The self is consequently focused on its physical aspects, i.e., intero- and/or exteroceptive self, while, at the same time, being locked-out from its mental self. This may account for the extreme degrees of ego-centeredness of ASD subjects: they can neither access their own self nor others’ selves on the mental level, which leaves them with no choice but to focus on the more physical layers of their own self, i.e., ego-centeredness. Whether such restriction to the intero- and exteroceptive layers of their self can also account for the often-observed abnormalities in sensory input processing with abnormally strong perceptual experiences [18,19,20,21,22,109,110] remains to be shown in the future.

### 4.4. Relationship to Other Theories I: Predictive Coding and the Hierarchical Model of Self

Predictive coding is about the prediction of an input and its degree of divergence from the actual input, i.e., the prediction error [111,112]. Importantly, there is hierarchy of predicted inputs and predictions within the brain itself. Specifically, higher levels provide a predicted input for the lower layers’ actual input: higher levels may predict the input processed in the next lower level, which, in turn, provides a predicted input for the next lower level and so forth. There is thus a nested hierarchy of predicted inputs and prediction error within the brain itself, an internal generative model, which is matched and compared with the hierarchy of external inputs stemming from body and environment [111].

This model of predictive coding has also been applied to ASD; one of the key proponents is Jacob Hohwy, who proposes that the internal generative model of the brain shows a flatter hierarchy in ASD [29]. Specifically, he proposes that the uppermost layer is thinner in ASD with lower number of nodes, while the lower layers of the internal generative model may, as compensation, be enriched with more nodes than in neurotypical subjects. Moreover, Perrykkad and Howhy (2019) assume that the upper layer provides a longer time and space scale than the lower layers, whose time–space scales are more restricted [29]. This means that prediction error minimization through predicted input focuses on short-term prediction, while long-term prediction is more or less neglected due to the sparsened most upper layer.

The autistic self is associated with more short-term features and details, as is well manifest in the sometime amazing autistic subjects’ tendency to pick up details that we, as neurotypicals, would overlook—this is one key feature of the weak central coherence theory (WCC; see below for details) [113,114,115,116]. However, conceived in a longer temporal perspective, the autistic self will lack temporal continuity and persistence—it becomes temporally fragmented and modular as it is “sliced up” into distinct self-fragments of different durations without temporal transitions or “glue” [29,93]: “In the case of the autistic self, this would mean that long-term invariances (as captured in the idea of personal identity over time and space) are more poorly represented than short-term, context-specific self-inferences.” [29] (p.17).

The temporal differences between upper and lower layers carry important implications for the model of self, which, in the predictive coding framework of Hohwy, occupies the role as hidden cause [29]. In ASD, the self, due to the disruption of the DMN and its longer timescales [117], is more based on short-term features of the lower layers and their higher variability as they are prone to and perturbed by the continuous external input—this concerns mainly sensory and interoceptive regions of the brain, like in the two lower layers accounting for the intero- and exteroceptive self in the hierarchical model of Qin et al. (2020) [58].

**Figure 8:**
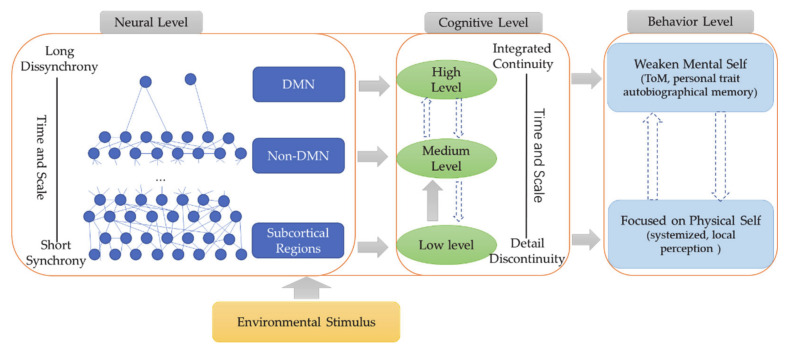
The altered hierarchy of the autistic self on neural, cognitive, and behavioral levels in ASD.

In contrast, weakening of the anterior and posterior midline DMN decreases the strength of the more long-term upper layer, which leaves the self without any psychological or mental stabilization independent of the ongoing perturbing inputs at the lower layers. Accordingly, we assume that the shift in the internal generative model with a weakening of the upper layer in Perrykkad and Hohwy (2019) converges well with her suggested HAS [29] (see Figure 8 and Table 3). The altered hierarchy of the autistic self may thus be characterized also in terms of temporal and spatial extension: the disruption of their mental self and its DMN leaves the autistic self with much more restricted time and space scales as limited to the ones of lower cortical and subcortical regions. Let us expand upon that in the following.

### 4.5. Relationship to Other Theories II: From Predictive Coding to Intrinsic Neural Timescales

Considered in this way, the changes in the autistic self may ultimately be traced to a shift from longer intrinsic neural timescales of the DMN to the shorter ones of cortical and subcortical non-DMN regions—there may thus be a temporal basis of the self-disturbance in ASD. Albeit tentatively, this is indeed in line with two recent papers showing abnormal intrinsic neural timescales in cortical and subcortical regions in ASD [118,119]. Reduced internal temporal continuity of self may be compensated for by increasing external temporal continuity in one’s action, such as by stereotypies, ritualization, rigidity, and obsessive-compulsive-like behavior [93], which decrease the prediction error and thereby provide internal temporal continuity [29].

Following the more externally oriented or ecological prediction, ASD subjects create their ecological niche in the external environment in terms of maximal stability and continuity: this helps avoiding surprises in the input in order to minimize their disruptive and unpredictable effects for the already temporally unstable short-term internal self—the authors speak of a highly inflexible precision of prediction errors in autism account (HIPPEA), which can be conceived more of an ecological rather than cognitive model of ASD [120,121,122]. Together, this amounts to a reversal between internal and external temporal continuity in ASD compared to neurotypical subjects: while neurotypical subjects use the internal temporal continuity to stabilize their self across time, ASD subjects may be forced to rely more on external temporal continuity to establish temporal continuity of their self.

Such temporal (and also spatial) approach to the lost hierarchy of self in ASD, i.e., HAS, is well compatible with the assumption of ASD being a temporo-spatial processing disorder that can be traced to the brain’s dysconnectivity and dissynchrony [123]. The DMN shows decreased connectivity, which reflects decreased synchronization between anterior and posterior midline DMN—that, as explicated above, may lead to the loss of temporal integration and longer timescales on the psychological level, such as, for instance, in mental self and episodic simulation. Loss of longer timescales on the neural level of DMN may thus transform into corresponding loss of temporal continuity of self and temporal expansion during episodic simulation—loss of longer timescales thus provides a “common currency” of neural and psychological levels [124,125] of the self and its internally oriented cognition in ASD (see Figure 8 and Table 3). However, to support such hypothesis, future studies may want to combine the investigation of neural dynamics like autocorrelation window with the measurement of psychological dynamics in episodic simulation, mental self, and mind-wandering.

### 4.6. Relationship to Other Theories III: Temporal Extension of Weak Central Coherence Theory

One highly influential theory is the WCC mainly promoted by Frith and Happe [113,126,127,128]. The key point here is that ASD subjects lack the coherence of linking different contents to their respective context; autistic subjects may thus be able to excel in mathematics or engineering but have problems of grasping more context-related subject matters, including languages. They often think about things in the smallest parts and their full details but are not able to see them in their larger spatiotemporal context—they cannot perceive or cognize the larger picture, that is, the “wood for the trees”. There is thus a disbalance between local and global cognitive activity, with the latter being impaired while the former may be abnormally strong. While there has been some controversy about the WCC [129,130], there is also strong support for the predominantly local–regional character of both perception and cognition in ASD [131,132,133].

We here would like to link the WCC to the reduction of the mental self and its above-mentioned deficit in internally oriented cognition. The anterior and posterior midline DMN mediate episodic simulation, the virtual expansion of the present time point into both past and future [76,78]. The various events or contents that physically have taken place at one specific timepoint may thereby be detached and shifted to another purely virtual time point in the subjects’ imagination, i.e., mental time travel. External inputs may thus be integrated within such internally ongoing virtual expansion of time of present into past and future.

As detailed above, DMN hypoactivity and connectivity in ASD subjects may make impossible such virtual expansion of inputs, which then restricts their cognition to (temporally) local levels of the present while blocking more global extension into past and future. Accordingly, we conceive the WCC in a temporal and dynamic way, which allows us to frame the relation of local vs. global perception/cognition as relation between shorter and longer intrinsic neural timescales (see Table 3). Future studies may want to probe some of the typical WCC tasks in conjunction with a temporal dynamic analysis on both psychological and neural grounds (see Watanabe et al., 2019 [118] and Damiani et al., 2019 [119] for a first step).

### 4.7. Relationship to Other Theories IV: Theory of Mind Mechanisms (TOMM) and Their Temporal Basis

Yet another influential theory is the Theory of Mind Mechanisms (TOMM) [5,6,7]. Here, the pronounced social cognition deficits are supposed to be based on deficits of imagining another person’s minds on a cognitive level [6,134]. That is complemented by a more precognitive and prereflective account in phenomenology where changes in different levels of intersubjectivity (primary precognitive, secondary-cognitive, and tertiary-metacognitive) are assumed in ASD (see Nilsson et al., 2019, 2020 for overview [91,92]).

We suppose that both cognitive and precognitive changes in intersubjectivity can ultimately be traced to the issue of temporal (and spatial) scales: if the range of the self’s internal temporal scales is rather limited and short, the person will have difficulties encoding and attuning to the much larger scales of its external environmental context—the subject will thus be socially isolated. In contrast, a wide range of different temporal scales, including both short and long, may allow the internal self to much more strongly connect and synchronize with its external environment—the subject will be well integrated. Accordingly, the limited range of timescales with the potential loss of especially the longer ones of the DMN may restrict the autistic self’s capacity to attune and synchronize with the large range of different, i.e., short and long, timescales of its environment—social isolation may be traced to temporal isolation (see Table 3).

## 5. Conclusions—A Hierarchical and Spatiotemporal Approach to the Autistic Self

We reviewed the neural findings on the self in ASD. One key finding is decreased activity of anterior and posterior midline DMN regions during different self-specific tasks, thus indicating a domain-general DMN deficit. Additionally, anterior and posterior midline DMN regions show decreased functional connectivity among each during both rest and task states accompanied by abnormal connectivity to non-DMN regions, including frontal, temporal, subcortical, and cerebellum.

Drawing on a recently introduced multilayered nested hierarchical model of self [58], we propose that those with ASD suffer from a DMN-based deficit in the mental self, accounting for the weakened self-referentiality with decreases in both self-awareness and self–other distinction. ASD subjects are locked-out of their mental self. That, in turn, leads to a disbalance of the weakened if not absent mental self with the lower layers of self, the intero- and exteroceptive selves as variants of the physical self. The combination of a DMN-based abnormally weakened mental self with a non-DMN and subcortically based abnormally strengthened processing of intero- and exteroceptive self may account for the seemingly paradoxical co-occurrence of increased ego-centeredness and decreased self-referentiality in ASD, i.e., the paradox of self [26]. Together, this amounts to what we describe as Hierarhical Model of Autistic Self (HAS).

Reviewing the various findings and discussing different theories, we propose converging the HAS with a primarily temporal and spatial framework. Our review establishes that the spatial topography of DMN is altered in the brain of those with ASD and that these changes disturb the neural hierarchy of self with the disbalance of mental and physical layers of self. Initial findings suggest that these changes in DMN may go along with altered intrinsic neural timescales in the same regions [118,119]—if replicated and confirmed this would render the self-disorder in ASD a truly spatial and temporal disorder conforming to what has been recently introduced as “Spatiotemporal Psychopathology” [78,135,136].

Specifically, combined spatial and temporal DMN changes make it rather difficult if not impossible for the autistic self to extend its physical self in spatial and temporal regard and thereby to constitute its own self on the mental or psychological level. The autistic self is consequently limited to the smaller time and space scales of its physical self, i.e., intero- and exteroceptive self, as mediated by subcortical and cortical non-DMN regions. This restriction of the autistic self, including its internally oriented cognition to shorter timescales and less extended spatial scales, leaves it socially isolated from its temporo-spatially more extended and complex environmental context.

In sum, the autistic self is locked-out of the larger timespace scales of the DMN and its mental self while being limited to the smaller timespace scales of its subcortical and cortical non-DMN-based intero- and exteroceptive self, i.e., physical self. Such spatiotemporal approach to the autistic self does not only explain the self-paradox but also aligns well with and extends dominant theories of ASD like predictive coding and weak central coherence theory. We conclude that our hierarchical and spatiotemporal model of the autistic self, i.e., HAS, opens novel and more specific ways of therapeutic intervention to re-balance the altered spatiotemporally based neural hierarchy of self.

## Figures and Tables

**Table 1:** Characteristics and main findings of fMRI studies with self-relative tasks in individuals with ASD.

N	Authors	Participants	Stimulus	Neural Activation and Functional Connectivity
1	Sumiya et al., 2020	Adults (N = 31)	pseudo-interactive joke task (self-relevant social reward)	ASD < TD: arMPFC in Self vs. Baseline
2	Lai et al., 2019	Adults (Male = 29; Female = 28)	adjective personality traits (mentalizing vs. physical)	ASD < TD (males), but ASD = TD (females): vMPFC in self vs. others ASD < TD (males), but ASD = TD (females): right TPJ in mentalizing vs. physical tasks
3	Cynan et al., 2019	Adults (N = 15)	Adjective personality traits (past self, present self; close other)	ASD >TD: bilateral STG/TPJ, right Ins, PCC and left MTG in past-self vs. close other ASD = TD: bilateral STG/TPJ, right Ins, PCC and left MTG in present self vs. close other
4	Hashimoto et al., 2017	Adults (N = 26)	Adjective personality traits (first vs. third perspective)	ASD < TD (in first-perspective): left TPJ/IPL in self vs. other
				ASD < TD: FC between left IFG and bilateral FPC in third vs. first perspective
5	Kana et al., 2017	Adults (N = 15)	Adjective personality traits (self, teachers, and letter)	ASD < TD: left IFG and left IPL in self vs. letter; THA, caudate, CRE and cuneus in self vs. teacher; ASD = TD: MPFC in self vs. teacher ASD < TD: FC between right ACC and bilateral IFG, right MTG, and left-putamen; left ACC and left IFG and CER during self-processing.
6	Pfeifer et al., 2013	Children and adolescent (N = 18)	Adjective personality traits (social vs. academic)	ASD < TD: vMPFC, MCC and left AI in self vs. others; ASD = TD (youth): MPPC (PCUN, PCC, and RSC) in self vs. other
7	Lombardo et al., 2009	Adults (N = 29)	Adjective personality traits (mental vs. physical)	ASD < TD: MCC; ASD = TD: vMPFC in self vs. other ASD < TD: FC between vMPFC and PSC, frontal operculum/VPMC in self vs. other
8	Kennedy & Courchesne, 2008	Adults (N = 13)	Self-personality traits (internal vs. external)	ASD < TD: vMPFC/vACC in Self vs. others ASD = TD: dMPFC, left ANG, and RSC/PCC in others vs. self
9	Chiu et al., 2008	Adolescents (N = 12)	multitrust play game (self vs. other decision)	ASD < TD: MCC during making own decision
10	Morita et al., 2016	Adults (N = 14)	Emotion processing (embarrassment) in self-face recognition	ASD < TD: right OC including IOG in self vs. other ASD = TD: right IFG and Ins in self vs. other ASD < TD: FC between caudal ACC and MPFC in observation vs. unobserved;
11	Mortia et al., 2012	Adults (N = 15)	Emotion processing (embarrassment) in self-face recognition	For ASD, self > others in bilateral mid-IFG and THA, the left STG, right Ins, and ACC. ASD < TD: PCC in self vs. others; right Ins for both condition.
12	Silani et al., 2008	Adults (N = 14)	Self-emotion awareness (internal vs. external)	ASD < TD: MPFC, right ACC, left PCUN, left TP and CRE in internal vs. external awareness; left AI in viewing unpleasant vs. neutral pictures.
13	Hogeveen et al., 2020	Adolescent and Young adults (N = 47)	Episodic memory encoding tasks	ASD > TD: right HC in relational vs item-specific ASD = TD: bilateral MTL in Relational vs. item-specific ASD < TD: FC between right PHC and left HC; right PHC and left PCC; but ASD = TD between MTL and whole brain.
14	Cooper et al., 2017	Adults (N = 21)	Episodic memory tasks	ASD = TD: HC, PPC, MPFC in both encoding and retrieval vs. baseline. ASD < TD: LPFC in retrieval vs. baseline; ASD =TD: strength node of LPFC and PCC during retrieval phase; ASD < TD: strength node of HC, FC between HC-FPCN and HC-DMN during retrieval phase, but no group difference during decoding.
				HC and MPFC activity increased for both ASD and TD in successful vs. unsuccessful retrieval; no group difference in correlation between HC, MPFC, and PCC activation and successful retrieval/retrieval precision.
15	Gaigg et al., 2015	Adults (N = 13)	Episodic memory with words triplet task	ASD > TD: left IFG; ASD < TD: left posterior HC in successful encoding vs. baseline. ASD = TD: bilateral MTL in Remember vs. Known
16	Greimel et al.2012	Children and Adolescents (N = 13)	Episodic memory (social vs. non-social)	ASD < TD: right IFG, left MFG, right IPL in Recol–NoRecol Face (social), bilateral SMG/dMPFC in Recol–NoRecol House (non-social). ASD = TD in AMG, FFG, or HC activation in Recol–NoRecol House (non-social)
17	Failla et al., 2020	Adults (N = 46)	Heart beating counting	ASD = TD: bilateral Ins.
18	Okamoto et al., 2018	Adults (N = 18)	Self and other hand pictures	ASD < TD: MPFC (including left MFG and ACC) in third vs. first perspective;
19	Uddin et al., 2008	Children (N = 12)	Self-face	ASD = TD: right IFG, MFG, PrCG, ANG, SPL, bilateral LOC, and FFG in self-face.

Note: MPFC—ventral medial prefrontal cortex; vMPFC—ventral medial prefrontal cortex; arMPFC—anterior rostral medial prefrontal cortex; dMPFC—dorsal medial prefrontal cortex; LPFC—lateral prefrontal cortex; IFC/IFG—inferior frontal cortex/inferior frontal gyrus; MFG—middle frontal gyrus; FPC—frontopolar cortex; VPMC—ventral premotor cortex; IPL—inferior parietal lobule; MPPC—medial posterior parietal cortex; PPC—posterior parietal cortex; SPL—superial parietal lobule; PSC—primary somatosensory cortex; PrCG—precentral gyrus; OC—occipital cortex; IOG—inferior occipital gyrus; LOTC—lateral occipitotemporal cortex; LOC—lateral occipital cortex; TPJ—temporo-parietal junctions; STG—superior temporal gyrus; MTG/MTL—middle temporal gyrus/lobe; TP—temporal Pole; FFG—fusiform gyrus; ACC—anterior cingulate cortex; PCC/PCG—posterior cingulate cortex/gyrus; MCC—middle cingulate cortex; PCUN—precuneus; RSC—retrosplenial cortex; ANG—angular gyrus; AI—anterior insular; Ins—Insular; HC-hippocampus; PHC—parahippocampal cortex; AMG—amygdala; THA—thalamus; CRE—cerebellum; SMG—superior medial gyrus; FPCN—fronto-parietal task control network (including lateral prefrontal and inferior parietal cortices); DMN—default-mode network; FC—functional connectivity.

**Table 2:** Characteristics of resting state fMRI studies in individuals with ASD.

N	Authors	Year	N of ASD	Method	N	Authors	Year	N of ASD	Method
1	Shi et al.	2020	11-ADO	seed-based	50	Kennedy et al.	2006	12-ADU	seed-based
2	Keehn et al.	2020	50-CHI and ADO		51	Cherkassky et al.	2006	57-ADU	
3	Bednarz & Kana	2019	37-CHI		52	Francis et al.	2019	13-CHI and ADO	ICA-based: ROIs
4	Iidaka et al.	2019	311-CHI, ADO and ADU		53	Igelström et al.	2016	26-ADO	
5	Odriozola et al.	2019	53-CHI, ADO, and ADU		54	Washington et al.	2014	24-CHI and ADO	
6	Margolis et al.	2019	17- CHI and ADO		55	Von dem Hagen et al.	2013	15-ADU	
7	Lawrence et al.	2019	16-ADO		56	Walsh et al.	2019	49-ADU	ICA
8	Reiter et al.	2019	44-CHI and ADO		57	Yao et al.	2016	31-CHI and ADO	
9	Olivito et al.	2018	10-ADU		58	Cerliani et al.	2015	166-CHI, ADO, and ADU	
10	Huang et al.	2018	39-ADU		59	Jann et al.	2015	17-CHI and ADO	
11	Voorhies et al.	2018	111-CHI		60	Nomi & Uddin	2015	72-CHI, ADO, and ADU	
12	Fisherman et al.	2018	55-CHI and ADO		61	Bos et al.	2014	27-CHI and ADO	
13	Pascual-Belda et al.	2018	75-CHI and ADO		62	Uddin et al.	2013	20-CHI	
14	Yan et al.	2018	531-CHI, ADU, and ADO		63	Assaf et al.	2010	15-CHI and ADO	
15	Traynor et al.	2018	30-CHI and ADO		64	Kozhemiako et al.	2020	194-CHI	ReHo, ALFF
16	Hogeveen et al.	2018	49-ADO and ADU		65	Keehn et al.	2019	57-CHI and ADO	
17	Yang & Lee	2018	40-ADO		66	Nair et al.	2018	147-CHI and ADO	
18	Olivito et al.	2017	8-ADU		67	Dajani & Uddin	2016	53-CHI, ADO, and ADU	
19	Joshi et al.	2017	15-ADO and ADU		68	Paakki et al.	2010	28-ADO	
20	Lin et al.	2017	20-CHI and ADO		69	Chen et al.	2020	37-CHI	graph theory
21	Burrows et al.	2016	53-CHI, ADO, and ADU		70	Itahashi et al.	2014	46-ADU	
22	Hoffmann et al.	2016	78-ADU		71	Li et al.	2018	15-CHI	Mix method
23	Abbott et al.	2016	37-CHI and ADU		72	Floris et al.	2018	360-CHI, ADO, and ADU	
24	Guo et al.	2016	30-ADO		73	Itahashi et al.	2015	50-ADU	
25	Shen et al.	2016	43-CHI		74	Martino et al.	2014	360-CHI, ADO, and ADU	
26	Green et al.	2016	28-ADO and ADU		75	Roll et al.	2020	394-ADO and ADU	dynamic
27	Alaerts et al.	2016	82-CHI, ADO, and ADU		76	Kupis et al.	2020	17-CHI	
28	Chien et al.	2016	37-ADO		77	Raatikainen et al.	2020	20-ADU	
29	Rausch et al.	2016	20-ADO and ADU		78	Mash et al.	2019	62-CHI and ADO	
30	Zhou et al.	2016	209-ADO and ADU		79	Fu et al.	2019	170- CHI, ADO, and ADU	
31	Kleinhans et al.	2016	25-ADO and ADU		80	He et al.	2018	28-CHI	
32	Farrant & Uddin	2016	20-CHI and 15-ADU		81	Guo et al.	2018	209-ADO and ADU	
33	Khan et al.	2015	28-CHI and ADO		82	Besseling et al.	2018	125-CHI, ADU, and ADO	
34	Doyle-Thomas et al.	2015	58-CHI and ADO		83	Falahpour et al.	2016	76-CHI, ADO, and ADU	
35	Fisherman et al.	2015	35-CHI and ADO		84	Holiga et al.	2019	841-CHI, ADO, and ADU	degree centrality
36	Yerys et al.	2015	22-CHI		85	Lee et al.	2017	329-CHI, ADO, and ADU	
37	Nair et al.	2015	37-CHI and ADO		86	Long et al.	2016	64-CHI, ADO, and ADU	
38	Chien et al.	2015	40-CHI and ADO		87	Di Martino et al.	2013	56-CHI	
39	Fisherman et al.	2014	40-ADO		88	Oldehinkel et al.	2019	265-CHI, ADO, and ADU	whole-brain
40	Lynch et al.	2013	20-CHI		89	Anteraper et al.	2019	24-ADU	
41	Delmonte et al.	2013	28-ADO and ADU		90	Chen et al.	2018	29-CHI	
42	Murdaugh et al.	2012	13-ADU		91	Cheng et al.	2017	394--CHI, ADO, and ADU	
43	Wiggins et al.	2011	39-CHI and ADO		92	Duan et al.	2017	91-ADO	
44	Ebisch et al.	2011	14-CHI and ADO		93	Lee et al.	2016	458-CHI, ADO, and ADU	
45	Di Martino et la.	2011	20-CHI		94	Cheng et al.	2015	418-ADO	
46	Weng et al.	2010	16-ADO		95	Tyszka et al.	2014	19-ADU	
47	Monk et al.	2009	12-ADU		96	Gotts et al.	2012	31-ADO and ADU	
48	Kennedy & Courchesne (a)	2008 ^a^	12-ADU		97	Anderson et al.	2011	40-ADO and ADU	
49	Kennedy & Courchesne (b)	2008 ^b^	13-ADU						

Note: CHI—children; ADO—adolescents; ADU—adults; ReHo—regional homogeneity; ICA—independent component analysis; ALFF—amplitude of low-frequency fluctuation; Mix method—including at least two methods of ReHo, ALFF, DC, ICA, or graph theory.

**Table 3:** Summary of the relationship between the Hierarchical Model of Autistic Self (HAS) and other theories.

HAS			PC	WCC	TOMM
DMN	INTs	Self			
1.Hypoactivity of DMN; 2.Hypoconnectivity between anterior and posterior DMN; 3.Hypoconnectivity between anterior DMN and non-DMN;	Reduced internal temporal continuity	Locked-out mental self and internally oriented cognition	Lack of long-term prediction(internal-orientation)	Overlook large pictures (global)	Hard to encode large scale of external social stimulus
1.Hypoconnectivity betwwen DMN and subcortical regions; 2.Hyperconnectivity between DMN and cerebellum	Increased external temporal continuity	Increasing Ego-centeredness	Focused on short-term prediction (external-orientation)	Pick up details (local)	Tend to encoding short scale of external social stimulus

Note: HAS—Hierarchical Model of Autistic Self; DMN—default-mode network; INTs—intrinsic neural timescales; PC—predictive coding; WCC—weak central coherence; TOMM—Theory of Mind Mechanisms.
